# Data capture in bioinformatics: requirements and experiences with Pedro

**DOI:** 10.1186/1471-2105-9-183

**Published:** 2008-04-10

**Authors:** Daniel Jameson, Kevin Garwood, Chris Garwood, Tim Booth, Pinar Alper, Stephen G Oliver, Norman W Paton

**Affiliations:** 1 School of Chemistry, Manchester Interdisciplinary Biocentre, The University of Manchester, 131 Princess Street, Manchester, M1 7DN, UK; 2 School of Computer Science, The University of Manchester, Oxford Road, Manchester, M13 9PL, UK; 3 NERC Centre for Ecology and Hydrology, Mansfield Road, Oxford, OX1 3SR, UK; 4 Faculty of Life Sciences, The University of Manchester, Michael Smith Building, Oxford Road, Manchester, M13 9PT, UK; 5 Department of Biochemistry, University of Cambridge, Sanger Building, 80 Tennis Court Road, Cambridge, CB2 1GA, UK

## Abstract

**Background:**

The systematic capture of appropriately annotated experimental data is a prerequisite for most bioinformatics analyses. Data capture is required not only for submission of data to public repositories, but also to underpin integrated analysis, archiving, and sharing – both within laboratories and in collaborative projects. The widespread requirement to capture data means that data capture and annotation are taking place at many sites, but the small scale of the literature on tools, techniques and experiences suggests that there is work to be done to identify good practice and reduce duplication of effort.

**Results:**

This paper reports on experience gained in the deployment of the Pedro data capture tool in a range of representative bioinformatics applications. The paper makes explicit the requirements that have recurred when capturing data in different contexts, indicates how these requirements are addressed in Pedro, and describes case studies that illustrate where the requirements have arisen in practice.

**Conclusion:**

Data capture is a fundamental activity for bioinformatics; all biological data resources build on some form of data capture activity, and many require a blend of import, analysis and annotation. Recurring requirements in data capture suggest that model-driven architectures can be used to construct data capture infrastructures that can be rapidly configured to meet the needs of individual use cases. We have described how one such model-driven infrastructure, namely Pedro, has been deployed in representative case studies, and discussed the extent to which the model-driven approach has been effective in practice.

## Background

### Why Data Capture Matters

Although many biologists are shielded from the intricacies of the field, the use of bioinformatics tools is a day-to-day reality for many. Modern high-throughput technologies yield substantial volumes of complex data that need to be stored, indexed and analysed. Effective storage and annotation of such data is imperative as, for example, the burgeoning field of Systems Biology emphasises the need to cross-reference and analyse results from multiple experiments and multiple sources.

The nature of biological research and the evolution of technology have resulted in myriad bespoke systems for data capture, storage and retrieval as reflected in the increasing number of databases documented in The Molecular Biology Database Collection [1] – 115 in 2000, 548 in 2004, and 968 in 2007. Yet this represents only a fraction of the data resources currently in use in the life sciences, since it only counts those that are publicly accessible. Internally, research groups and institutions rely on a wide range of systems and architectures, ranging from the archiving of lab books to well-designed experimental data repositories. As a result, numerous sites undertake data capture tasks so that experimental and derived data can be analysed and archived. Despite this ubiquity, data capture requirements, tools and techniques receive rather little direct attention in the bioinformatics literature.

### Types of Data

Biology is a broad field and, as a result, there are a wide variety of data types that may need to be captured and annotated. Nonetheless, biological data can be grouped into three major categories:

*1) Primary data*, which are generated directly by experiments. These may be recorded electronically in output files from instrumentation, by hand in lab books or as images. Examples include micrographs, fitness measurements from different organisms, sequence data, spectroscopy results, and microarray image files.

*2) Secondary data*, which are derived from primary data, are the results of some form of analysis that has been performed on the primary data. These include things like protein structures, phylogenetic trees, normalised gene expression values and protein identifications. Sometimes secondary data are generated from a single piece of primary data, but often they are the results of analyses involving primary or secondary data from many sources.

*3) Metadata*, which may relate to primary data or secondary data or both, provides detail and context to the data. Examples include experimental protocols, references to standard experimental materials, experimenter details, times and dates, keywords and bibliographic references.

Although data resources may combine these different types of data in different ways, the following scenarios are common:

1) Primary data with associated metadata, e.g. GenBank [2].

2) Secondary data with associated metadata, e.g. pFam [3].

3) Primary data and directly derived secondary data with associated metadata, e.g. SGD [4].

### Requirements of Data Capture Tools

Requirements of data capture tools vary depending on the context within which the data is to be captured, the nature of the data, and the use to which it is to be put. However the following requirements have been identified in several contexts:

R1. Import and export of data in appropriate formats.

R2. Manipulation and update of existing records.

R3. Checking of integrity of captured data.

R4. Callable by, or able to call on, other applications.

R5. Able to use controlled vocabularies for annotation.

R6. Able to selectively re-use existing data in new entries.

### Existing Data Capture tools for Bioinformatics

This section reviews several representative data capture solutions for bioinformatics. These have been selected as they fulfil the following critera:

1) They are publicly available.

2) They are used for capturing rich descriptions.

3) They represent a wide range of functionalities.

4) They include offerings from the major international biological data centres (EBI-EMBL, NCBI).

The extent to which the solutions examined fulfil the requirements listed above is summarised in Table 1. We note that not all requirements are relevant to all contexts, so a solution may be effective for a given application without supporting all the requirements.

**Table 1:** Requirements of data capture tools

Requirement	Pedro	Sqeuin	maxDLoad2	ArrayExpress	MAGE-TAB	PRIDE
R1	✓	✓	✓	✓	✓	✓
R2	✓		✓		✓	✓
R3	✓	✓	✓	✓		✓
R4	✓					
R5	✓	✓	✓	✓		✓
R6	✓	✓	✓	✓		✓

### Sequin and Webin

**Figure 1:**
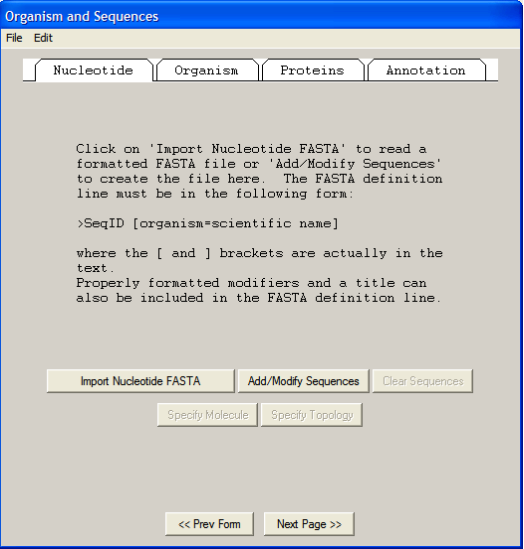
**Sequin data capture tool**. The user is led through import of data and subsequent annotation stepwise. This results in a file suitable for submission to GenBank, EMBL or DDBJ.

Sequin and Webin [2] support data capture and submission for the NCBI's GenBank sequence database, and are capable of marking up DNA, mRNA and protein sequences in a format suitable for submission to the GenBank, EMBL and DDBJ databases. The user is led through a series of steps to construct the annotated file that will ultimately be uploaded (Figure 1). The software also allows visualisation of sequence data before uploading. A high level of context-sensitive help is available, and is displayed alongside the data entry window.

Although short sequences are reasonably straightforward to annotate by hand, larger sequences (for example complete genomes) require a different approach. To support this, Sequin allows the bulk import of sequence features through the use of a tab-delimited file. While this does not afford the same level of validation as the software interface, it speeds the submission of entries that may contain thousands of individual annotations.

### maxdLoad2

**Figure 2:**
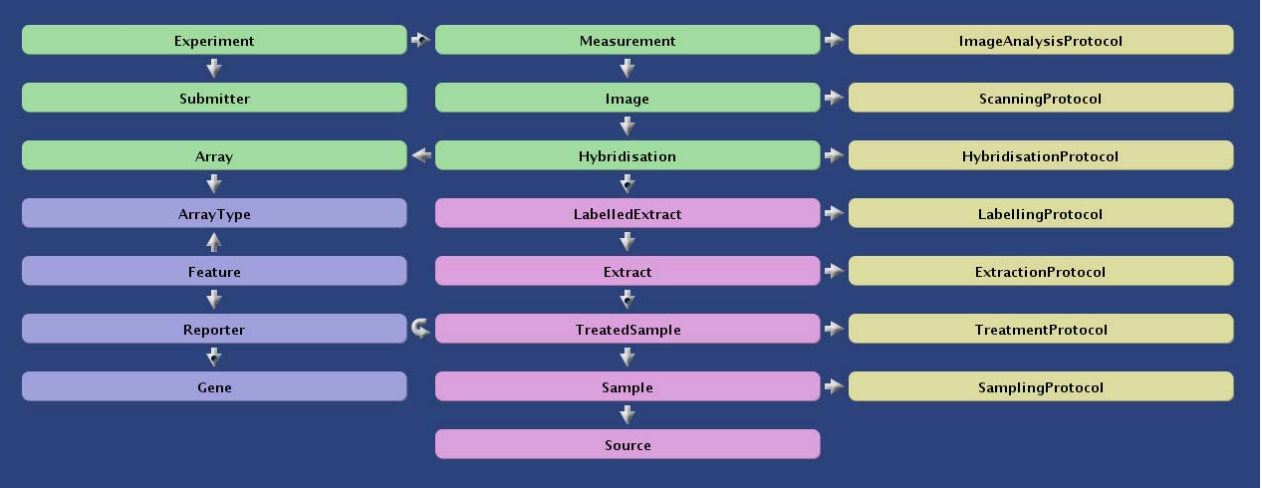
Relationship between entities in the maxdLoad2 data model.

maxdLoad2 [5] is a data-loader/annotator and repository for microarray datasets that, once captured, can be manipulated and analysed using maxdView (Figure 2).

Microarray datasets from experiments that are to be published often have to be annotated to the level of detail specified by the MIAME [6] (Minimum Information to Annotate a Microarray Experiment) standard, and there is generally a requirement that the annotated experiments are submitted to a public repository (e.g. GEO [7] or ArrayExpress [8]) before publication. maxdLoad2 allows the export of annotated data in MAGE-ML [9] format, which may be read into the ArrayExpress database. Although free text may be used to provide annotation up to MIAME standards, maxdLoad2 provides a logical route through each level of annotation, which ensures that the standard is reached.

Much of the data entered (e.g. array types) can be re-used in future annotations, thereby reducing the amount of work that needs to be undertaken when a subsequent experiment is loaded. Although it takes time to annotate a simple experiment using the point and click interface of maxdLoad2, it is also possible to bulk upload annotations in a spreadsheet format and then refine the annotations within the software.

### ArrayExpress

ArrayExpress is a public repository for microarray experimental data [8]. It provides both a web-based and a Java application for the annotation and upload of experimental data to the database. Again, these lead the user through a series of steps, by the end of which they have annotated their data to the level required by MIAME.

The above solutions are representative of the diverse collection of bespoke data capture interfaces found in bioinformatics. They are essentially "Wizards", leading the annotator through a pre-defined path at the end of which they will have generated a dataset suitable for storage in the desired repository. This achieves the aim of simplifying the loading of data to a point where scientists can typically perform the task with minimal training, but can result in certain annotation procedures becoming long-winded and repetitive; hence the oft-provided facility to bulk upload data in a spreadsheet or tab-delimited format.

### Generic Infrastructures

Generic data capture infrastructures are desirable as they can, at least in principle, be rapidly applied to new data capture contexts. While a bespoke solution, given appropriate resources, should be able to support the requirements of specific tasks and user communities directly, the ability to instigate a data capture regime quickly when a need is identified can be essential to keeping the data capture infrastructure in step with experimental practice, while also reducing development costs.

Spreadsheets are generic data capture and analysis tools. They permit the entry and editing of tabular data, and can output these data in a tab-delimited format that can be quickly parsed into databases. In addition, modern spreadsheets can be programmed to check that the data entered satisfies certain completeness and integrity constraints. Furthermore, the look and feel of the sheet may be adapted to isolate the user from its inner workings; for example, by only allowing data to be entered in specific cells. Spreadsheets have the added advantage that many biologists use them on a regular basis to tabulate and analyse data. With this in mind, there have been recent efforts to produce data-capture spreadsheets for specific purposes.

### PRIDE

PRIDE is the PRoteomics IDEntifications database at the EBI [10]. Submission of data in an XML format conforming to a model defined as an XML Schema is encouraged. A spreadsheet known as Proteome Harvest has been developed that supports the generation of PRIDE-XML.

The spreadsheet works with Microsoft Excel, and makes extensive use of Microsoft's Visual Basic Scripting language to generate XML documents. The user is guided through a number of spreadsheet tabs where the requisite data to build the XML document is entered. Additionally, when appropriate, values for fields can be extracted from a remote, controlled vocabulary database to ensure that values are drawn from consistent terminologies. For example, when detailing biological source samples, ontology terms may be retrieved from NEWT (Taxonomy), the BRENDA Tissue Ontology and the Cell Type Ontology to provide a complete and controlled description of that sample.

**Figure 3:**
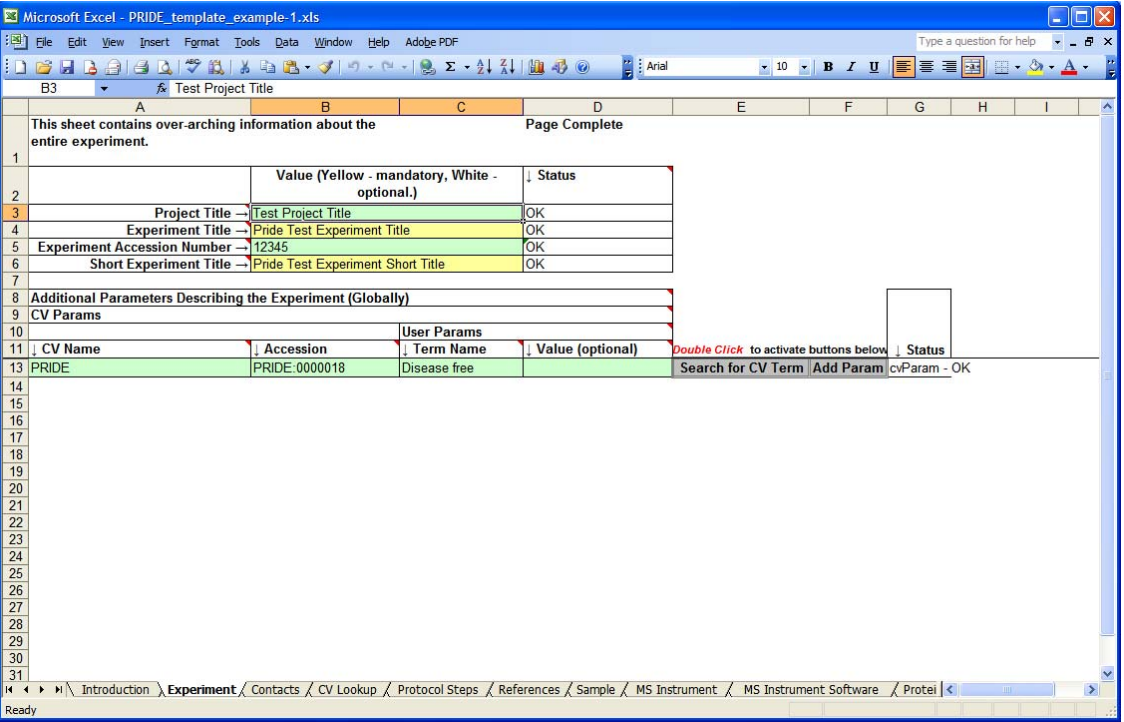
**Proteome Harvest Spreadsheet**. Macro functions allow searching for ontology terms and provide validation for input data.

The Proteome Harvest Spreadsheet is essentially a generic tool (i.e. Excel) that has been configured to support a specific task by software plug-ins that generate PRIDE-XML from a collection of custom data entry forms (Figure 3).

### MAGE-TAB

MAGE-TAB is a standard format for annotating microarray datasets to the MIAME standard using spreadsheets or tab delimited files [11]. It relies on four separate files to define an experiment:

#### 1) Investigation Description Format

This is a tab-delimited file that gives general information about the experiment performed, including contact details, references and free-text descriptions of the protocols used.

#### 2) Array Design Format

This describes the array used and the sequences associated with individual array locations. It may alternatively provide an accession number linking it to a pre-defined array already located in a public repository.

#### 3) Sample and Data Relationship Format

This tab-delimited file describes the relationships between the samples, arrays, and data generated by the experiment.

#### 4) Raw and processed data files

These are typically native format data files generated by the array imaging software. Alternatively there is a "data matrix" tab-delimited format in the MAGE-TAB specification.

This approach is at the other end of the spectrum from Proteome Harvest in the sense that it uses the spreadsheet solely as format to be adhered to. The drawback with this method of data capture is the lack of validation in the procedure – as the files are simply text and generated using which ever text editor the user desires, there can be no checks for consistency in, for example, the Sample and Data Relationship file. This makes it more likely that annotation errors will be introduced when capturing a complicated dataset.

Effective data capture is a recurring need in the biological sciences. As a result, there are many bespoke solutions with overlapping capabilities, and several activities are building on spreadsheets as a configurable infrastructure. However, as yet, there is limited practical experience with configurable data capture infrastructures, and the development of bespoke infrastructures (such as Sequin and Webin for GenBank) continues to be a significant drain on bioinformatics resources.

## Results

### Model-driven data capture in Pedro

The Pedro software [12] was originally developed to support data capture in the PEDRo proteomics model [13], in a context where it was anticipated that standard models and ontologies would evolve rapidly. Thus, rather than developing a bespoke application that would need regular changes to its code base, a model-driven approach was adopted in which the code base is independent of the structure of the data to be captured.

In model-driven software development, aspects of the behaviour of a system are configurable using models [14]. In Pedro, there are two models: (i) a *domain model*, represented in XML Schema, which describes structure of the data that is to be captured, and against which the resulting XML file must validate; and (ii) a *configuration model*, which characterises aspects of the behaviour of the application. In Pedro, the configuration model defines the context sensitive help (relevant information is dynamically displayed when the mouse passes over specific form fields and labels), where ontologies are to be used for populating specific elements in the domain model, which plug-ins are to be used and from where they can be obtained, and what validation is to take place in addition to that supported directly by the domain model.

Pedro is part of a family of model-driven tools that includes the Pierre system for accessing biological databases [15]; Pedro is used to edit the models that drive Pierre applications. Where the developers of a model-driven system anticipate differences between the requirements of different applications, and can capture those differences using a model, the development of a new application reduces to the provision of a new model. Where a new application requires highly specialised behaviour, such as the extraction of data from a proprietary file format, such behaviours may be supported by plug-in software components, for which extensibility points are provided by the model-driven architecture.

A data capture application in Pedro involves the following components:

#### 1) A model of the data to be captured

the structure of the data is represented as an XML Schema [16].

#### 2) An optional collection of plug-in software components

where specialised behaviour is required by the application, for example connecting to a remote controlled vocabulary server or reading from a proprietary file format, software components can be provided that implement these capabilities. These reside in a library jar file which is specific to the domain model in use.

#### 3) A model of the behaviour of the system

the model of the behaviour of the system (the *configuration model*). This model resides in a configuration file which is itself created and revised using Pedro.

#### 4) The Pedro software

Given 1) to 3), the Pedro software provides an interactive user interface with which to capture data conforming to the model in 1).

Below we describe in more detail how the model is used to generate forms and how the functionality of the tool may be extended through the use of ontologies, plug-ins and validation services.

### Form Generation

Pedro captures hierarchical documents which consist of records containing fields which may contain further records. Forms are the fundamental method by which a user interacts with Pedro to capture data. Pedro's forms are driven by user defined XML Schema Definitions (XSDs) which describe the records and fields that may appear in a document. Information about additional labels, form buttons and which fields they are associated with is read from the configuration file discussed above.

**Figure 4:**
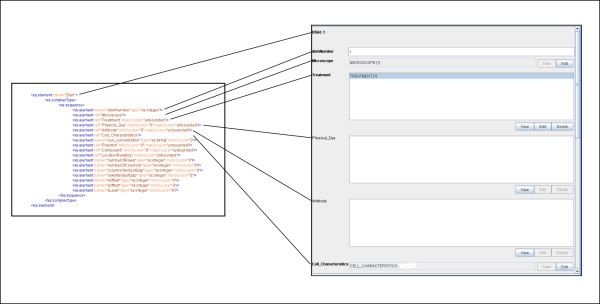
**Pedro uses an XML Schema to generate forms for data capture**. Here the direct mapping between schema and form elements is illustrated. List boxes are displayed where an element may have multiple instances or simple text boxes where only one instance is allowed. Compulsory fields are rendered in bold type.

Pedro renders forms using information combined from the schema and configuration file. Figure 4 shows a section of schema that may be used to describe part of a microscopy experiment and how the elements in the schema map directly to form fields rendered on screen.

Possible form fields may be:

• Edit Fields – these may be Text Fields, URI Fields and Radio Buttons (grouped and ungrouped). Attached to these are any form comments, tool tips, help links defined in the configuration file.

• List Fields – these are lists of records that are children of the current record.

### Data Import/Export

Pedro is able to directly import data from files in its own native format, from XML files that conform to the current schema and also from tab delimited text files. This latter feature enables data that may have been captured or recorded in a spreadsheet tabular format to be rapidly imported into records within an XML document. When performing this operation, for any record in the document, columns within the file may be mapped to specific fields whilst multiple lines relate to multiple records.

Before finalisation of a document it may be saved in Pedro's native format for later editing; however when exporting to XML the document is tested for validity against the model schema and cannot be saved until it conforms.

### User Plug-ins

Without any additional coding, Pedro is able to generate forms from a schema, capture data, and output that data as an XML file that conforms to the original schema definition. This may be sufficient for some basic data capture applications, however to extend functionality plug-ins can be implemented by the developers who are configuring Pedro for use in a specific domain. The plug-ins may provide, for example, database access, data analysis and data import or export functions.

**Figure 5:**
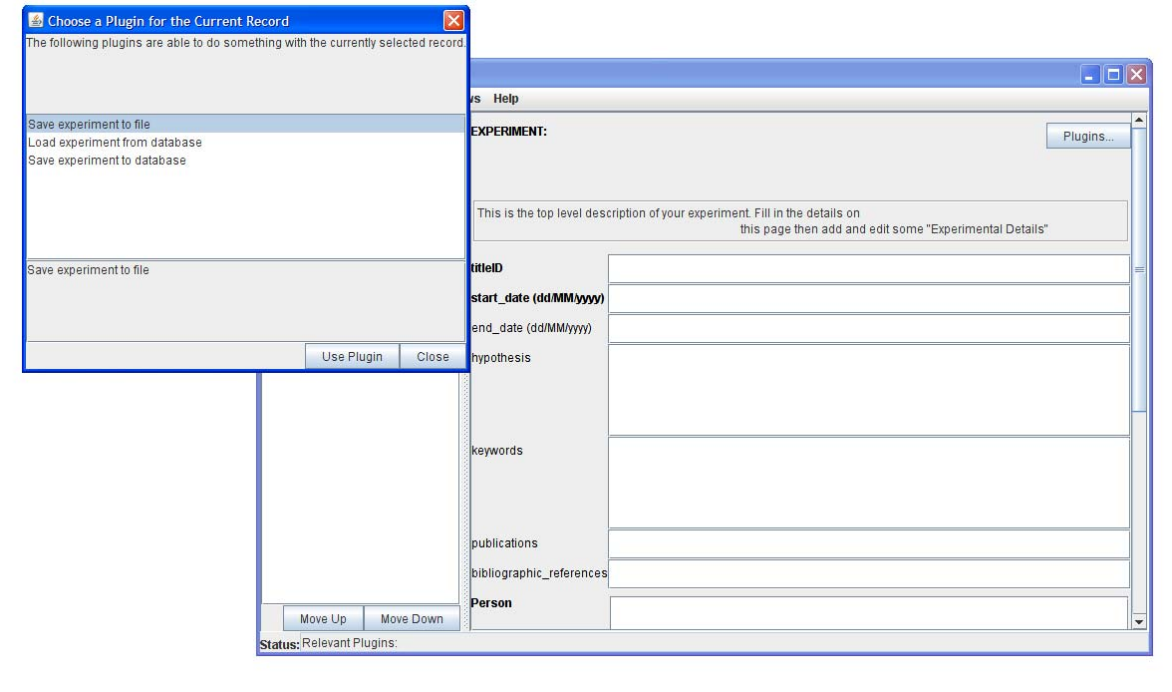
**Using a Pedro plug-in appropriate to the current record**. The top level record for the Cell Imaging annotation document has three different plugins (Save or Load from database and Save directly to File) associated with it which may either read from or manipulate the complete document structure.

The plug-ins themselves are written in Java and implement the PedroPlugin interface. The records and fields associated with a particular plug-in are defined by the Configuration File. When the data entry tool is running, plug-ins may appear in different places. Document-level plug-ins appear in one or more of the menus in the menu bar. If, during document editing, plug-ins are associated with the current record type, then a "Plug-ins." button is displayed in the main form (Figure 5). If plug-ins are associated with a field, the "Plug-ins." button appears at the end of the form field. Plug-ins have access to all of document data structures within Pedro, and can manipulate them independently of the user interface. This can be particularly useful when parsing foreign file formats into Pedro or enabling Pedro to interact directly with an external database. The use of plug-ins in a production environment is discussed in the Cell Imaging case study below.

### Ontology Services

An ontology service allows end-users to mark up a form field with terms that come from a controlled list of terms. Although its scope of effect is a single form text field, it may use other information about the current form, the user or document to help constrain the choices of terms it provides to the viewer. From a developer's perspective the ontology services comprise two parts, Ontology Source and Ontology Viewer. Terms are derived from the Ontology Source and presented to the user by the Ontology Viewer.

The Ontology Source for a particular field is defined within the configuration editor and manifests itself as a Java class that implements the OntologySource interface. A list of available OntologySource classes and interfaces is shown in Table 2, and by extending these or implementing a new OntologySource classes it is possible to utilise ontologies from a variety of sources (local or remote) unconstrained by format.

**Table 2:** Pedro Ontology Sources

Class/*Interface*	Function
*OntologySource*	Implementing classes provide ontology terms to an OntologyViewer.
*TreeOntologySource*	Extends OntologySource but implementing classes provide ontology terms that can be organised as a tree structure.
*DictionaryDescriptionSupport*	Implementing classes provide a definition for OntologyTerms.
*ImageDescriptionSupport*	Implementing classes can provide images based on an identifier.
*URLDescriptionSupport*	Implementing classes can provide a link to a webpage.
SingleColumnTextSource, *OntologySource*	An ontology source that reads terms from a text file that contains a single column of ontology terms.
AbstractTreeOntologySource, *TreeOntologySource*	A class to manage a tree of terms. This is extended in the classes below.
TabIndentedTextSource	Extends AbstractTreeOntologySource. This is an ontology source that reads terms from a tab-indented text file.
XMLOntologySource	Extends AbstractTreeOntologySource. This is an ontology source that reads its terms from a bespoke XML format.

**Figure 6:**
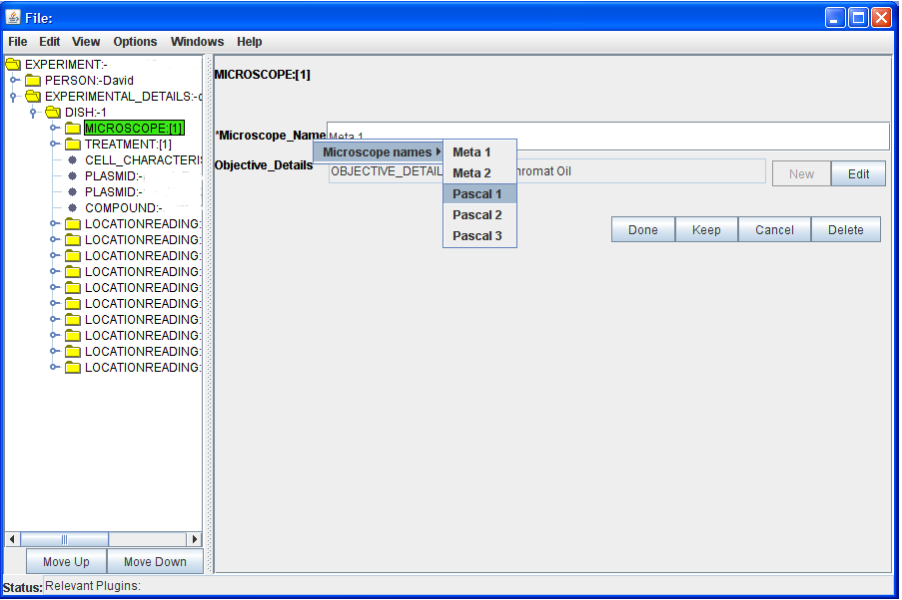
**Utilisation of an ontology**. Here a simple list type ontology is used to annotate details of Microscope Name in a record. Increasing the number of available ontology terms in a simple list will result in it being rendered in multiple sublists.

Ontology sources may also implement other interfaces, which allow Pedro to make a decision about which ontology viewer to use. For example, an ontology source which implements the TreeOntologySource interface will be able to provide Pedro with the information necessary to render that ontology as a tree, and hence Pedro will do as such. Again it is possible to implement new viewers by implementing the OntologyViewer interface or extending the existing viewers (Table 3). In Figure 6 we show the use of a simple ontology to control the names of microscopes available for use in the document being edited.

**Table 3:** Pedro Ontology Viewers

Class/*Interface*	Function
*OntologyViewer*	Implementing classes can render a set of ontology terms.
DefaultOntologyViewer, *OntologyViewer*	This is the default viewer used to render terms provided by ontology sources. Depending on the interfaces (Table 2) provided it can render the ontology terms in a variety of different ways:
	• Simple list
	• Tree
	• With dictionary definitions panel
	• With image thumbnail panel
	• With webpage panel

### Validation Services

Validation is extremely important for ensuring data integrity, and Pedro supports validation services which can affect a field, record or entire document. Field and record validation services are triggered whenever the user attempts to commit changes to the current record. Many of the validation constraints are implicitly defined in the XML Schema (field types, number of instances); however the facility exists to extend these with user defined validation classes which may then be specified in the configuration file.

Pedro's document-level validation services are triggered when users try to export a data set to a final submission format or when they use the "Show Errors" feature in the View menu.

Field-level validation services are intended to identify problems in the value of an edit field or with the composition of child records found in a list field. Record-level validation services are intended to identify field values which are legitimate when considered in isolation but are wrong when considered in combination with other field values. For example, within the microscopy experiment annotations, a form could have fields with values such as "start_date = 10/2/07" and "end_date = 4/2/07" which would form an illegal combination of values. Document-level validation services are intended to identify errors that appear in disparate parts of the same data set. A list of available validation service interfaces is shown in Table 4.

**Table 4:** Pedro Validation Service Interfaces

Interface	Context	Role
*ListFieldValidationService*	Field	Implementing classes provide a validation service for list fields that is triggered whenever the user explicitly activates "Show Errors" or when the current data file is exported to a final submission format.
*EditFieldValidationService*	Field	Implementing classes provide a validation service for edit fields that is triggered whenever the user explicitly activates "Show Errors" or when the current data file is exported to a final submission format.
*RecordModelValidationService*	Record	Implementing classes provide a validation service that is activated whenever the user tries to commit changes to the current record.
*DocumentValidationService*	Document	Implementing classes provide a validation service that is triggered whenever the user explicitly activates "Show Errors" or when the current data file is exported to a final submission format.

Pedro is a generic but highly configurable data capture tool which has allowed it to be deployed in the variety of scenarios we describe below.

### Case Studies

Here, we present three use-cases that have made use of Pedro. These examples, from different projects and laboratories, illustrate how data capture requirements encountered in practice can be addressed using the model-driven approach.

### Metadata Capture for Cell Imaging

This project has investigated gene function through high-throughput image analysis of living cells [17]. Using microscopy, fluorescently labelled proteins are tracked in real time as they move within cells. The experiments conducted allow a single microscope to observe a large number of fields in a single run. The archiving and indexing of these rich data sets requires a database to store the results of microscope runs along with associated descriptive metadata. Capturing and consolidating these data involves parsing and integrating output from multiple sources to conform to a schema that makes extensive use of controlled vocabularies. To support this functionality, a data capture infrastructure must support requirements R1, R2, R3, R5 and R6, as listed previously.

**Figure 7:**
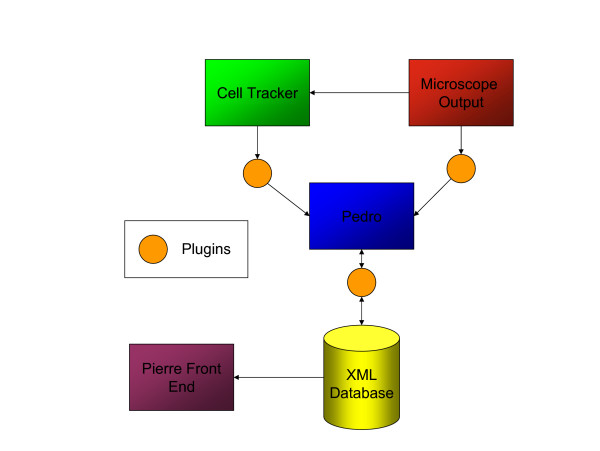
Organisation of the data capture and storage architecture for the cell imaging project.

The principal components in the data capture and storage infrastructure are illustrated in Figure 7. To encourage consistent annotation of data, it is important to minimise both the complexity and amount of manual annotation that must be performed. In this example, much of the information needed to describe the experiments comes from other software systems. Experimental metadata from the microscope is initially stored in a proprietary format, which is parsed and mapped into the domain model by a Pedro plug-in. The experimental metadata includes details about the microscopy equipment used, locations recorded, the time points at which data were collected, lasers used and their settings. Images from the microscope are analysed by Cell-Tracker [18], which extracts features from the images; these features are integrated with the experimental metadata using a second plug-in. Experimentalists use the interactive interface to provide additional experimental metadata, for example on the sample studied, and a third plug-in stores the data in the Tamino native XML database [19].

Table 5 provides a complete list of the plug-ins employed; the experiment import and export plug-ins allow the direct submission of the experiment to the database, as well as the retrieval and updating of records (Figure 5).

**Table 5:** Plug-ins for the Beacon Database

Plug-in	Function
Import/Export	These plug-ins allow the import and export of "Dye", "Plasmid", "Characteristic", "Person" and "Experiment" records from the database.
Equipment Import Plug-in	Reads a microscope database file and populates the record based upon this.
Spotted Import Plug-in	Reads a spotter program file and associated microscope database file and populates a record based upon these.
Spot Export Plug-in	Allows the export of a single spot record to a file.
Tracker Import Plug-in	Reads the XML output from Cell Tracker and adds this to a record.
Excel Import Plug-in	Reads the Excel output from Cell Tracker and adds this to a record.

Pedro's use of an XML Schema as the domain model, coupled with the Tamino XML DBMS [20], allows for validation of the generated document before submission to the database. The same schema is implemented in both locations, and thus ensures that model constraints are enforced consistently in different parts of the architecture. Further integrity of the data is ensured by using plug-ins to support experiment annotation using predefined XML entities stored on the database server. In this situation, an alternative methodology would be to employ Pedro's ontology features as described below.

Several features of the model-based approach have been important to this project. In particular, the model of the domain has gone through several iterations, to reflect changes in experimental practice and in the experimentalists' understanding of what it was appropriate to archive. The fact that changes to the model can be reflected immediately in the interface has supported an iterative development process where end users are able to quickly feed back on changes to the model, and further modifications and updates can be rapidly distributed to the end users without any modifications to their installations. Furthermore, support for plug-ins has enabled the interactive data entry features to be used in conjunction with several existing software systems in a distributed environment.

The end user experience of Pedro has, by and large, been positive. An initial deployment with laboratory-based biologists has seen its effective use for data capture with minimal training. As issues of clarity in the forms presented have become apparent, these have been addressed through use of annotations displayed in the form text, and the users are now annotating complete experiments to the standards required for the database.

### Use of the Pedro ontology services in the EnvBase data catalogue

EnvBase is a high-level, project-centric catalogue of datasets created and maintained by the NERC Environmental Bioinformatics Centre (NEBC). Due to the varied nature of projects recording data in the system, and the rapidly evolving nature of research in environmental genomics, a flexible approach was required. It was decided to represent catalogue entries as XML documents, and to employ a range of free and open source software solutions – Perl, PostgreSQL, GenQuery and Apache – to implement the catalogue. Pedro was chosen as an editor for the catalogue of XML documents as it was easy to configure, runs standalone on platforms that support Java, while combining XML editing and validation with access to ontology services. Overall, to support this application, a data capture infrastructure must support requirements R1, R2, R3, R4 and R5, as listed previously.

The use of the ontology services in EnvBase is mainly confined to word lists. Both single-column lists (for example, to select the funding programme under which the work took place) and tree lists (for example, to select the repository within which a particular data holding resides) are used. An example of the latter is to select the type of data being catalogued; a hierarchy of data types was discussed and agreed within the data centre, and the master copy of this file is stored in a CVS repository. A disadvantage of this approach is that, because data may be annotated on many different machines, changes to any ontology file must be distributed to all these machines. An alternative arrangement for networked machines would be to use a plug-in that loads and caches terms from a server, removing the need for manual updates.

One situation where the basic ontology services proved insufficient was for browsing the NCBI taxonomy database, which has the following features:

• The taxonomy is large, with over 235,000 nodes.

• An organism is only put into the taxonomy if it is represented by some accession in the nucleotide sequence database and, therefore, species are continually being added.

• Each taxon has a canonical unique name within the database. Many species have one or more pseudonyms, and these names may be used by the researchers or in the literature, but we must record the official NCBI name.

The NCBI taxonomy is available as a flat file, suitable for loading into a relational database, or via a web services interface. For the reasons given above, particularly in relation to the size of the taxonomy, the standard ontology browsers within Pedro were insufficient. Thus a plug-in was developed, known as TaxInspector, for browsing a locally held copy of the database. TaxInspector retrieves and loads the flat files from NCBI into the local database, and then allows direct browsing over the taxonomy tree, searching over taxon names, common names and pseudonyms, and a quick access list to some of the most common species. When a taxon is selected, details of the selection are shown, as well as the full path from the root of the tree to that node.

**Figure 8:**
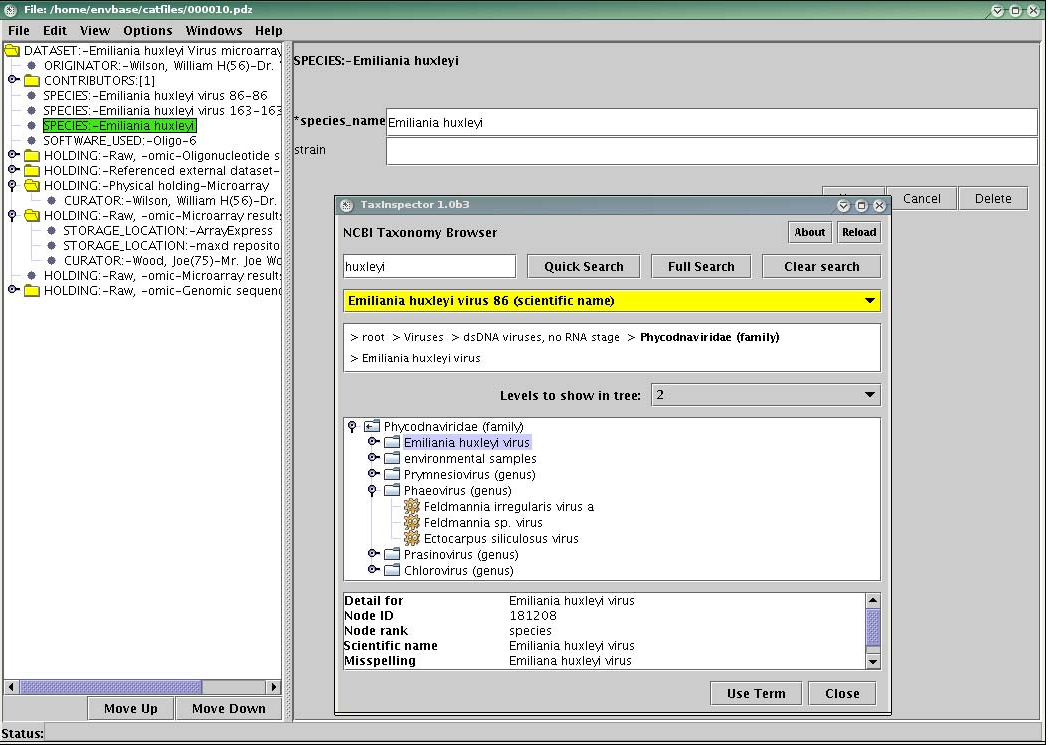
TaxInspector being called from Pedro.

TaxInspector can be run as a standalone Java application, but the ontology interface provided by Pedro made it straightforward to have the program run as a plug-in. When a user right-clicks on the "species_name" heading, the TaxInspector GUI appears. Once the desired taxon has been located, the user clicks on the "Use Term" button to insert the selection into Pedro. TaxInspector is shown in Figure 8.

The user group for Pedro in EnvBase consists of a small central team of curators and, periodically, members of the projects whose results are recorded in the catalogue. The impression is that, although there is an initial learning curve, the tool has been well received, with little training required. However, users do need to go through an initial familiarisation process. Pedro has been designed principally for use with complex data sets; as a result, it contains quite rich functionality and can seem complicated compared with typical online data entry forms, such as those that capture conference registration information. For example, Pedro allows data to be captured in different orders and supports the saving of partial data sets, so validation is less straightforward than in linear data entry systems, where all validation at one step is completed before moving on to the next step. In Pedro, some validation information, for example on mandatory fields, is always visible and users can perform global validation checks. However, different users may prefer different approaches to validation, and the presence of explicit functionality to request validation is an example of a feature that must be learned, and for which an effective mode of operation must be developed by users.

### Pedro as a ^my^Grid Workbench Plug-in

The ^my^Grid project [21] is developing service-based middleware and applications to support biological *in silico *experiments. *In silico *experimentation requires the integration of heterogeneous, disparate and autonomous bioinformatics resources, i.e. data and analysis tools, available on the web. The ^my^Grid environment allows scientist to discover resources, orchestrate them into stored workflows, enact these workflows, and manage and publish the results.

In ^my^Grid, Pedro has been used as part of resource discovery [22]. The number of bioinformatics resources available to the scientist is large (~3000) and increasing rapidly. These resources, generally exposed via web services, normally provide only limited descriptions of their functionality, their expected inputs and the outputs they produce. As a result, finding suitable resources for inclusion in a workflow poses a challenge. To address this, ^my^Grid provides an ontological model for representing the bioinformatics domain and service properties, over which a registry has been developed that allows storage and querying of service descriptions. As part of this framework, Pedro has been used as an annotation tool to help ^my^Grid users create service descriptions. Overall, to support this application, a data capture infrastructure must support requirements R1, R2, R3, R4 and R5, as listed previously.

To ease the overall service annotation process for users, Pedro has been integrated into the ^my^Grid workbench environment, rather than being used as a stand-alone tool. As part of the integration, the Pedro ontology services have been extended to support RDFS (Resource Description Framework Schema [23]) ontology sources. As Pedro uses XML Schema to describe domain models, XML is the sole serialization format supplied with Pedro. As a result, where the data are to be stored other than by using file-based XML, a plug-in must be developed that interfaces with the required data repository.

The model-driven approach and the generic nature of Pedro were exploited at the exploratory stages in the development of the service discovery framework. During this period, the discovery system was iteratively prototyped over a changing model, and was used to evaluate the service description model. In this setting, as in the cell imaging example, iterative development was supported by automatic user interface generation.

Pedro has been used by two categories of users within ^my^Grid, namely the scientists performing the *in silico *experiments and the bioinformatics domain experts/curators that specialize in building ontologies and service descriptions. Both groups of users, particularly the non-experts, found Pedro's display of a service description intricate in certain aspects, e.g. the tree view directly supports the tree structure in the model, and doesn't provide model-driven grouping of related elements to provide custom displays. This reflects a design feature in Pedro whereby there are no display models as are found in some other model-driven user interface development environments (e.g. [24]).

## Discussion

While the specifics of data capture vary from task to task, the underlying requirements outlined in the Introduction are common to most of the situations encountered when Pedro has been applied in the life sciences. Pedro goes some way to fulfilling all of these requirements:

R1. *Import and export of data in appropriate formats*. Pedro imports and exports XML, includes support for tab-delimited files, and plug-ins can be used to export to other formats.

R2. *Manipulation and update of existing records*. Pedro supports both creation and modification of documents described using the domain model.

R3. *Checking of integrity of captured data*. The model-driven approach allows captured data to be validated against the relevant XML schema, and plug-ins can be used to provide additional validation. This requirement is also supported through the use of ontology services.

R4. *Callable by or able to call to other applications*. Although Pedro can be used without plug-ins, data capture rarely takes place in isolation from other software tools, so the ability to call out to plug-ins, and be called down from as a plug-in, are supported, and used frequently.

R5. *Able to use controlled vocabularies for annotation*. Support for user-defined ontologies is a core feature, and plug-ins can be used to provide access to specialised ontology servers or browsers.

R6. *Able to selectively re-use existing data in new entries*. Pedro's models may specify data to be included in each record, removing the need for this to be entered manually.

Pedro occupies a niche, in which it seeks to support a wide range of data-capture tasks through a judicious mixture of configurable properties and plug-in services. This configurability enables Pedro to be deployed in diverse data-capture applications, while also providing sufficient functionality out-of-the-box to enable direct use in straightforward settings. As with other model-based systems, a balance has to be struck between the level of configurability provided and the complexity of the models required to provide that flexibility. In this context, Pedro seeks to constrain the complexity of model development by committing to a single type of domain model (i.e. XML schema), a single look and feel (i.e. a tree browser and form-based entry), and a single type of task (i.e. data capture). However, within those constraints, Pedro provides over 80 configuration options, thereby enabling significant enhancements to be made to default behaviours, and providing incremental tailoring to reflect user needs.

## Availability and requirements

The Pedro has been available from  since April 2004, since when it has been downloaded over 2,700 times. Pedro is distributed under the Academic Free License. The software has been tested on Windows-based platforms and currently depends on Java 1.4.

## Authors' contributions

DJ contributed to the development of the cell imaging application, wrote the first draft of the paper and made subsequent revisions. KG developed the Pedro software. CG contributed to development by documentation and testing. TB and PA provided text on their experiences using Pedro in EnvBase and ^my^Grid, respectively. SGO contributed application experience throughout the development of Pedro. NWP supervised the development of Pedro, and, together with SGO, refined the text.
